# Non-Invasive Assessment of Coronary Microvascular Dysfunction: From Flow Quantification to Clinical Decision Making

**DOI:** 10.3390/medicina62101954

**Published:** 2026-10-09

**Authors:** Panagiotis Theofilis, Athanasios Sakalidis, Kyriakos Dimitriadis, Paschalis Karakasis, Aggelos Papanikolaou, Panagiotis Iliakis, Aikaterini Vordoni, Panayotis K. Vlachakis, Panagiotis Tsioufis, Eleni Adamopoulou, Vasileios Panoulas, Konstantinos Aznaouridis, Konstantinos Tsioufis, Dimitris Tousoulis

**Affiliations:** 1 First Department of Cardiology, National and Kapodistrian University of Athens, Hippokration General Hospital of Athens, 11527 Athens, Greece; asakalidis@gmail.com (A.S.); dimitriadiskyr@yahoo.gr (K.D.); agepap25@otenet.gr (A.P.); panayiotisiliakis@gmail.com (P.I.); katerinavord@gmail.com (A.V.); vlachakispanag@gmail.com (P.K.V.); ptsioufis@gmail.com (P.T.); 99elenaadam@gmail.com (E.A.); conazna@yahoo.com (K.A.); ktsioufis@gmail.com (K.T.); drtousoulis@hotmail.com (D.T.); 2 Harefield Hospital, Royal Brompton and Harefield Hospitals, Guy’s and St Thomas’ NHS Foundation Trust, London SE1 7EH, UK; vasileios.panoulas2@nhs.net; 3 Second Department of Cardiology, Hippokration General Hospital, Aristotle University of Thessaloniki, Konstantinoupoleos 49, 54642 Thessaloniki, Greece; pakar15@hotmail.com

**Keywords:** coronary microvascular dysfunction, myocardial blood flow, myocardial flow reserve, positron emission tomography, cardiovascular magnetic resonance, echocardiography, SPECT, CT perfusion, INOCA, ANOCA

## Abstract

Coronary microvascular dysfunction (CMD) is a major mechanism of myocardial ischemia and adverse cardiovascular outcomes in patients with angina or dyspnea, both in the absence and in the presence of obstructive coronary artery disease. Because the coronary microcirculation cannot be visualized directly with routine angiography, diagnosis depends on physiological assessment. Non-invasive imaging has moved beyond relative perfusion analysis toward quantification of resting and hyperemic myocardial blood flow and flow reserve. Positron emission tomography (PET) remains the most extensively validated non-invasive method and provides robust absolute flow measurements with strong prognostic evidence. Quantitative stress cardiovascular magnetic resonance (CMR) is rapidly maturing, combining high-resolution perfusion mapping with ventricular and tissue characterization without ionizing radiation. Transthoracic Doppler echocardiography offers an accessible, radiation-free estimate of coronary flow velocity reserve, whereas myocardial contrast echocardiography can assess myocardial blood volume and replenishment kinetics. Dynamic cadmium-zinc-telluride single-photon emission computed tomography and stress computed tomography perfusion extend flow quantification to more widely available platforms, although standardization and outcome validation remain less developed. Across modalities, interpretation requires attention to the adequacy of vasodilator stress, resting hemodynamics, diffuse epicardial atherosclerosis, scar, and technical factors. A reduced flow reserve is not synonymous with isolated microvascular disease and cannot identify acetylcholine-provoked vasospasm. This review provides a clinically oriented framework for selecting, performing, interpreting, and reporting non-invasive CMD testing; examines the evidence, strengths, and limitations of each modality; and defines situations in which invasive coronary function testing remains necessary. Future progress will depend on cross-platform standardization, automated quality control, disease- and sex-specific reference ranges, and prospective trials linking imaging-defined endotypes to effective treatment strategies.

## 1. Introduction

Coronary microvascular dysfunction (CMD) defines the impaired ability of the coronary microcirculation to manage myocardial blood supply to metabolic demand. As CMD is frequently seen in contemporary cardiology practice, mainly in patients with angina or exertional dyspnea and no obstructive coronary artery disease (ANOCA/INOCA), its clinical importance is undisputed [1,2,3]. Within these syndromes, CMD may represent one mechanism among several, including epicardial vasospasm, microvascular spasm, mixed vasomotor abnormalities, and diffuse non-obstructive atherosclerosis. CMD may also coexist with obstructive coronary artery disease or myocardial disorders. This distinction is clinically important because conventional vasodilator flow imaging predominantly assesses coronary vasodilator capacity and does not exclude vasospastic disease when flow measurements are normal.

CMD is clinically relevant because affected patients may experience persistent angina or exertional dyspnea, recurrent healthcare utilization, impaired quality of life, and an increased risk of adverse cardiovascular events. CMD is nevertheless frequently missed, since conventional coronary angiography assesses the epicardial lumen. Standard stress imaging, which is optimized to identify regional perfusion defects induced by flow-limiting epicardial stenoses, may also appear normal when flow is globally and relatively homogeneously reduced. As a result, symptoms may be ultimately attributed to non-cardiac causes, particularly in women, since a visually normal coronary angiogram may be interpreted as evidence that ischemia has been excluded. The resulting diagnostic gap is clinically relevant, since ‘non-obstructive coronary arteries’ is an anatomical, but not physiological, description.

The diagnostic landscape has changed with the availability of methods that can accurately quantify myocardial blood flow (MBF) at rest and during vasodilator stress [4], such as positron emission tomography (PET) and quantitative perfusion cardiac magnetic resonance (CMR) [5,6,7]. Transthoracic Doppler echocardiography is another method of coronary flow velocity reserve (CFVR) estimation in the left anterior descending artery, while myocardial contrast echocardiography (MCE) evaluates microvascular blood volume and replenishment [8]. Dynamic cadmium-zinc-telluride (CZT) single-photon emission computed tomography (SPECT) and computed tomography perfusion (CTP) are extending physiological assessment to additional platforms [9,10].

Recent international guidelines and consensus documents have incorporated non-invasive flow assessment into the evaluation of suspected CMD, while emphasizing that persistent symptoms and suspected vasomotor disorders may require invasive coronary function testing [5,6,7]. This review focuses on how non-invasive tests should be used in clinical practice rather than treating them as interchangeable technical measurements. We first define the physiological signal provided by each test, then review modality-specific evidence, practical advantages and limitations, clinical applications, and the residual role of invasive testing.

## 2. Rationale and Core Concepts in Non-Invasive CMD Assessment

The coronary microcirculation comprises pre-arterioles, resistance arterioles, capillaries, and venules below the spatial resolution of routine coronary angiography. Resistance arterioles are the principal regulators of flow and respond to various signals. At rest, autoregulation maintains myocardial perfusion across a range of perfusion pressures. During exercise or pharmacological vasodilation, coronary resistance falls and flow increases several-fold. However, this adaptive response may become impaired, leading to CMD. Several mechanisms have been implicated, including arteriolar remodeling and luminal narrowing, capillary rarefaction, perivascular fibrosis, endothelial dysfunction, exaggerated vasoconstriction, impaired smooth muscle relaxation, or microvascular spasm, among others [1,2,3].

The central non-invasive quantities are resting MBF, hyperemic or stress MBF, and their ratio. PET, CMR, dynamic SPECT, and dynamic CT can estimate MBF in milliliters per minute per gram of myocardium. Myocardial flow reserve (MFR), often termed myocardial perfusion reserve (MPR) in CMR literature, is calculated as stress MBF divided by rest MBF. Doppler echocardiography instead measures CFVR as a stress-to-rest ratio. When epicardial vessel caliber is stable, the velocity ratio is a useful surrogate for volumetric flow reserve. MCE derives myocardial blood volume and microbubble replenishment velocity, whose product approximates tissue blood flow.

These indices are related but not identical. MFR may be the most appealing since the ratio partially normalizes interindividual differences and technical scaling. However, it can be low for two fundamentally different reasons: inadequate hyperemic flow or abnormally high resting flow. The first pattern is more consistent with reduced vasodilator capacity caused by diffuse atherosclerosis, structural CMD, or insufficient pharmacological stress. The second is frequently encountered with tachycardia, hypertension, obesity, anemia, pain, anxiety, or increased myocardial workload. A patient with stress MBF within the expected range but high resting MBF may therefore have a reduced ratio without severe structural microvascular disease. Conversely, a normal ratio can camouflage reduced absolute flow when both rest and stress values are proportionally low. Therefore, the components of the ratio should always be reported, rather than using MFR as an isolated binary variable [5,6,11,12]. Accordingly, when absolute quantification is available, the flow-reserve ratio should always be interpreted together with the absolute resting and hyperemic MBF values and the accompanying coronary and myocardial phenotype.

Resting flow is closely linked to rate-pressure product, but simple linear correction is imperfect. Although a correction may be informative when the resting hemodynamic state is clearly abnormal, both corrected and uncorrected values should be interpreted with caution since correction formulas assume relationships that may not apply in certain scenarios, such as in heart failure, left ventricular hypertrophy, or severe valvular disease. Hyperemic flow is also influenced by age, sex, tracer, kinetic model, software, scanner characteristics, vasodilator type, caffeine exposure, and comorbidities. For this reason, a universal numerical definition of CMD does not apply across all platforms.

A second conceptual distinction is between endothelium-independent and endothelium-dependent function. Adenosine, regadenoson, and dipyridamole predominantly test arteriolar smooth muscle vasodilation through adenosine pathways. Reduced hyperemic flow or flow reserve therefore indicates impaired vasodilator capacity but is not sufficient for diagnosing endothelial dysfunction. As a result, non-invasive tests may reveal ischemia during spontaneous or provoked symptoms, but they are unable to distinguish the underlying mechanism.

A third distinction concerns the epicardial coronary arteries. Global MFR integrates resistance across the entire coronary circulation. A low value may arise from isolated CMD, diffuse non-obstructive atherosclerosis, multivessel obstructive disease, or combinations of these conditions. Regional reductions favor focal epicardial disease but may also occur with heterogeneous microvascular dysfunction or prior infarction. A diagnosis of isolated CMD therefore requires reasonable exclusion of flow-limiting epicardial disease. Even after obstructive disease has been excluded, diffuse plaque burden can impair flow and has independent prognostic significance. The most clinically useful interpretation is thus phenotypic rather than dichotomous: anatomy, regional perfusion, global and regional flow, scar, ventricular structure, and stress adequacy should be combined for a well-justified conclusion.

Finally, non-invasive flow measurements are myocardial rather than purely coronary signals (Table 1). Fibrosis, infarction, cardiomyocyte hypertrophy, high wall stress, extravascular compression, and elevated filling pressure can reduce measured tissue perfusion even without a primary arteriolar disorder. While this may appear as a flaw, it may explain why impaired flow reserve has prognostic implications across multiple diseases. It does mean, however, that the report should state the clinical context and avoid implying a single histopathological mechanism from one numerical threshold.

## 3. Positron Emission Tomography

PET is the most established non-invasive technique for quantifying MBF [4,6,12]. Dynamic acquisition begins with tracer arrival in the blood pool and continues through myocardial uptake, allowing time–activity curves to be fitted with tracer-specific kinetic models [6,13]. Common tracers include rubidium-82, nitrogen-13 ammonia, and oxygen-15 water [6,13]. Their extraction fractions, production requirements, image characteristics, and kinetic behavior differ [14]. The longer-lived fluorine-18 perfusion tracer flurpiridaz has expanded the prospect of regional distribution and exercise-capable PET, although implementation and outcome data are still developing [5,14].

A complete PET flow study provides rest and stress relative perfusion images, ventricular function, coronary calcification or attenuation CT information, segmental and global MBF, and MFR [4,6,12]. The interpretation should begin with technical quality: patient motion, attenuation–emission misregistration, tracer bolus integrity, count-rate saturation, segmentation, spillover correction, and adequacy of vasodilator stress [12,15]. Caffeine, methylxanthines, inadequate drug delivery, severe hypotension, and competing pharmacological effects can blunt hyperemia [6,12,15]. A falsely low stress value should be suspected when the hemodynamic response is absent, symptoms are minimal, or the flow pattern is implausibly uniform in a patient with low pretest probability [5,6].

When relative perfusion is normal, a preserved global MFR generally confers low short-term risk and makes clinically important diffuse coronary vasodilator dysfunction unlikely [5,16]. A reduced global MFR with normal perfusion is the classic non-invasive pattern that raises concern for CMD, diffuse epicardial atherosclerosis, or both [5,6]. The absolute stress MBF helps refine this phenotype [5,17]. Reduced MFR accompanied by low hyperemic MBF is more consistent with impaired vasodilator capacity, whereas reduced MFR with preserved hyperemic MBF and elevated resting MBF suggests a high-rest-flow phenotype [5,17]. Conversely, an apparently preserved MFR should not be considered evidence of normal coronary physiology when both resting and hyperemic MBF are abnormally low. Thus, absolute flow and flow reserve provide complementary rather than interchangeable information. The distinction has prognostic and mechanistic implications and has been emphasized in recent PET consensus statements and contemporary phenotyping studies [5,17]. Regional flow reduction that corresponds to a perfusion defect suggests focal epicardial disease, but multivessel disease can produce balanced reduction with limited relative contrast [18,19]. Integration with coronary calcium, CCTA, or angiographic data is therefore essential [5,6,12].

PET flow values should be interpreted as continuous physiological measures rather than against a single universal diagnostic threshold, because absolute values vary with tracer, kinetic model, software, laboratory methodology, and patient characteristics [5,6,12]. For practical clinical interpretation, global MFR > approximately 2.0 is commonly regarded as preserved, values of 1.7–2.0 as mildly reduced, 1.2–<1.7 as reduced, and <approximately 1.2 as severely reduced when the study is technically valid. These categories are approximate interpretive ranges rather than biological cutoffs. Similarly, hyperemic MBF values below approximately 1.7–2.3 mL/min/g have been associated with abnormal physiology, although the applicable value depends substantially on tracer and methodology [6,13]. Accordingly, locally validated reference ranges and transparent reporting of the tracer, stressor, kinetic model, and software should take precedence over rigid application of a universal numerical threshold [5,6,12].

The evidence supporting PET is substantial. Large cohorts have shown that reduced MFR predicts cardiac death, myocardial infarction, and heart failure hospitalization beyond clinical variables, left ventricular function, and relative perfusion findings [16,19,20]. The risk relationship is graded rather than binary. Clinically, this means that patients with similar conventional perfusion findings may have substantially different risk according to MFR; in a prospective cohort of patients undergoing 82Rb PET, impaired MFR independently predicted hard cardiac events (HR 3.3) and major adverse cardiovascular events (HR 2.4), beyond conventional perfusion findings [19].

In patients referred for angiography, global flow reserve integrates the effects of focal and diffuse coronary disease and can modify the prognostic association between revascularization and outcome [18]. PET-defined impairment is also prognostically informative in women and men, in diabetes and chronic kidney disease, and in patients with cardiomyopathy or HFpEF [20]. These observations support the interpretation of MFR as a global marker of coronary circulatory health rather than a disease-specific test [5,18,19].

The major practical advantages and limitations of PET are summarized in Table 2. In brief, PET provides highly reproducible absolute flow quantification, robust assessment of diffuse or balanced physiological impairment, and strong prognostic validation [5,6,12,13,15], but availability, tracer-related infrastructure, radiation exposure, motion/attenuation artifacts, and model-dependent quantification remain relevant limitations. Importantly, PET cannot identify vasospastic endotypes and evidence that treatment based on a particular PET flow threshold improves outcomes remains limited [5,6,12,15,17,18,19].

For clinical reporting, a PET CMD report should state the indication; tracer and stressor; image and stress quality; relative perfusion; rest and stress global MBF; global MFR; relevant regional values; the pattern driving any low reserve, left ventricular function, calcium or CT findings; and the likely integrated interpretation.

## 4. Cardiac Magnetic Resonance

Stress perfusion CMR provides high-spatial-resolution assessment of first-pass gadolinium transit through the myocardium [7,10,11]. Conventional clinical interpretation is visual: a subendocardial or transmural stress-induced defect that persists for several cardiac cycles and exceeds a coronary territory threshold suggests ischemia [7,11]. CMD, however, may produce subtle circumferential subendocardial hypoperfusion or globally reduced signal augmentation that is difficult to recognize visually [7,21]. Semiquantitative indices, including myocardial perfusion reserve index (MPRI), compare the upslope of myocardial enhancement at stress and rest [7,21]. Fully quantitative techniques estimate absolute MBF by modeling the arterial input function and tissue enhancement and increasingly generate automated pixel-wise perfusion maps [11,22].

Quantitative CMR requires accurate measurement of both the arterial input and myocardial tissue curves. A concentrated contrast bolus can saturate the left ventricular blood-pool signal, so dual-bolus or dual-sequence strategies are used to preserve linearity [22,23]. Contemporary inline or near-inline pipelines perform motion correction, myocardial segmentation, arterial input function extraction, and pixel-wise kinetic modeling [17,22]. The resulting maps display stress MBF, rest MBF, and myocardial perfusion reserve, enabling global and regional assessment [11,17]. The 2025 Society for Cardiovascular Magnetic Resonance consensus statement provides a framework for acquisition, analysis, quality assurance, and reporting, reflecting the transition from single-center research toward multivendor clinical use [24].

Validation studies have compared quantitative CMR with PET and invasive indices such as fractional flow reserve and index of microvascular resistance [11,25]. Automated mapping can identify flow-limiting epicardial disease and demonstrate lower global stress MBF in patients with invasive evidence of microvascular dysfunction [11,25]. In women with suspected CMD, reduced MPRI has correlated with invasive coronary reactivity and predicted adverse events, although historical MPRI thresholds are sequence- and analysis-dependent [21]. Quantitative stress MBF and perfusion reserve provide incremental prognostic information even when conventional regional perfusion is visually normal [25]. Recent studies in ANOCA populations using automated workflows have shown compromised perfusion compared with healthy controls and have begun to define clinically implementable ranges [26,27].

CMR is particularly well suited to clinically complex patients because perfusion is only one component of the examination [7,25]. Cine imaging quantifies ventricular volumes and function; late gadolinium enhancement identifies infarction, replacement fibrosis, and myocarditis patterns; T1 mapping and extracellular volume estimate diffuse fibrosis or infiltration; T2 mapping can identify edema; and valvular or congenital abnormalities can be characterized [7,10]. This tissue context is essential when low flow reserve may be secondary to hypertrophy, scar, inflammation, or elevated wall stress [7,25]. High spatial resolution also makes CMR sensitive to subendocardial ischemia, an expected manifestation of microvascular disease and pressure-overload states [7,11].

The main practical limitation is incomplete standardization [7,10,27]. Absolute MBF varies with field strength, pulse sequence, temporal coverage, contrast dose, stressor, reconstruction, motion correction, segmentation, and kinetic model [10,22,23]. Published thresholds for abnormal stress MBF and perfusion reserve therefore cannot be treated as universal [21,25]. MPR values below approximately 1.5–2.0 have commonly been considered abnormal in published studies, but the applicable range varies according to acquisition sequence, field strength, reconstruction and kinetic model. Such values should therefore be regarded as indicative rather than definitive thresholds, with preference given to platform-specific validated reference ranges [10,27]. Visual, semiquantitative, and fully quantitative results should not be mixed in one threshold framework [7,10]. A report should clearly identify which method was used [10,27].

Technical and patient-related factors remain important sources of error. Dark-rim artifacts, motion, arrhythmia, inadequate breath-holding, and insufficient vasodilator stress may affect quantitative perfusion measurements, while caffeine or other anti-adenosinergic agents may produce false-negative stress responses [7,10,22]. Gadolinium use may be restricted in selected patients with advanced renal dysfunction, and claustrophobia, non-MR-compatible devices, or limited scanner availability may preclude testing [7,10]. These practical considerations are summarized together with the major advantages and limitations of CMR in Table 2.

A clinically useful quantitative CMR report should describe stress adequacy and image quality, visual perfusion findings, global and segmental stress MBF, rest MBF and myocardial perfusion reserve when available, the spatial distribution of reduced flow, ventricular function, late enhancement and mapping findings, and epicardial anatomy available from prior CCTA or angiography. In the absence of a regional defect and obstructive disease, globally reduced stress MBF and reserve support CMD. If the ratio is reduced primarily because rest flow is high, the report should state that the finding may reflect elevated resting workload rather than severe failure of hyperemic vasodilation.

## 5. Echocardiographic Assessment

Echocardiography is the most accessible radiation-free platform for physiological assessment, but the available techniques measure different aspects of the microcirculation [8,28]. Transthoracic Doppler echocardiography estimates flow velocity in an epicardial coronary artery, usually the distal left anterior descending artery, while MCE evaluates myocardial microbubble kinetics at the tissue level [8,28]. Standard wall motion stress echocardiography and myocardial strain are valuable for ischemia and ventricular mechanics but are relatively insensitive to homogeneous reductions in perfusion and should not be presented as direct CMD tests [8,28].

For transthoracic Doppler CFVR, color Doppler is used to identify the distal left anterior descending artery in the anterior interventricular groove [28,29]. Pulsed-wave Doppler records the characteristic biphasic coronary velocity signal, and peak diastolic velocity is measured at rest and during vasodilator stress. CFVR is the ratio of hyperemic to resting diastolic velocity [28,29]. Adenosine or dipyridamole is typically used, and regadenoson can be employed according to laboratory practice [28,29]. CFVR ≤ approximately 2.0 is commonly considered abnormal, although thresholds up to 2.5 have been used depending on the population, stressor, and acquisition methodology [8,28,29]. These values should therefore be regarded as practical interpretive thresholds rather than a universal definition of CMD.

The technique has meaningful clinical evidence. In the iPOWER study, CFVR measurement was feasible in a large cohort of women with angina and no obstructive CAD, and reduced CFVR was associated with cardiovascular risk factors and adverse prognosis [30,31]. Multicenter stress echocardiography cohorts have shown that CFVR adds prognostic information to wall motion and left ventricular contractile reserve across patients with suspected or known coronary disease [32,33]. The method is particularly appealing because it can be added to a conventional vasodilator stress echocardiogram without ionizing radiation or nephrotoxic contrast and can be repeated at relatively low cost.

Several limitations constrain generalization. The measurement is operator-dependent and requires specific training, optimal alignment with the coronary flow jet, and a stable sample volume. Feasibility falls with obesity, lung disease, tachypnea, and unfavorable acoustic windows. Most clinical laboratories assess only the left anterior descending artery, so the result may not represent the entire coronary circulation [28,34]. CFVR is a velocity ratio rather than absolute tissue flow and assumes that epicardial vessel area does not change materially [28,34]. It is affected by resting heart rate and blood pressure, epicardial stenosis in the measured vessel, prior infarction, collateral flow, and stress adequacy. Interobserver and test–retest variability are generally greater than with well-performed PET. A low LAD CFVR after exclusion of LAD stenosis supports impaired coronary vasodilator function but cannot by itself establish diffuse global CMD.

MCE uses intravenously administered gas-filled microbubbles that remain within the vascular compartment [35]. During continuous low mechanical index imaging, a high-energy pulse destroys microbubbles in the myocardium and their subsequent replenishment is recorded [35]. The plateau intensity reflects microvascular blood volume, the rate of replenishment reflects microvascular velocity, and the product approximates MBF [35]. Vasodilator stress can therefore reveal reduced augmentation of blood volume, velocity, or flow [35]. MCE has the conceptual advantage of assessing the entire myocardium and can identify diffuse subendocardial abnormalities that are missed by wall motion analysis [33,35].

Clinical adoption of quantitative MCE has been limited by acoustic artifacts, attenuation, microbubble destruction–replenishment methodology, contrast availability and reimbursement, and dependence on acquisition and post-processing expertise. Absolute values are less standardized than PET, and robust multicenter outcome thresholds for isolated CMD are lacking [8,35]. Nevertheless, studies in patients with non-obstructive coronary anatomy demonstrate that MCE can identify impaired microvascular perfusion and may add mechanistic information. MCE is best considered a specialized option in experienced centers rather than a universal substitute for PET or quantitative CMR.

When echocardiography is chosen, reporting should distinguish CFVR from MCE-derived perfusion and should avoid the generic phrase ‘coronary flow reserve’ without specifying the signal. A Doppler report should include the artery assessed, stressor and dose, rest and stress peak diastolic velocities, CFVR, signal quality, and whether obstructive disease in that artery has been excluded. MCE reporting should state the contrast agent, acquisition protocol, whether analysis was qualitative or quantitative, and the regional or global pattern of perfusion abnormality [35,36].

## 6. SPECT Myocardial Perfusion Imaging

Conventional SPECT is based primarily on relative tracer uptake. It remains effective for regional ischemia but may underestimate diffuse or balanced reductions because the best perfused region is used as the internal reference [9,37]. Dedicated CZT cameras have higher count sensitivity and temporal resolution, permitting dynamic imaging during tracer first pass and estimation of global and regional MBF and MFR [9,37]. This development is clinically important because SPECT infrastructure is more widely available than PET.

The WATERDAY study demonstrated the feasibility of dynamic technetium-99m sestamibi CZT-SPECT flow quantification against oxygen-15 water PET and invasive fractional flow reserve [37]. Subsequent studies and technical reviews have refined acquisition, input-function correction, and kinetic modeling and have reported associations between reduced SPECT-derived MFR and adverse outcomes [9,38]. An MFR around or below 2.0 is frequently used as an indicative abnormal range; however, CZT-SPECT values remain strongly dependent on camera geometry, tracer extraction, reconstruction, and software. Accordingly, no universal cross-platform cutoff should currently be applied, and locally validated ranges are preferable.

Dynamic SPECT has important constraints. Technetium tracers show non-linear myocardial extraction at high flow, which compresses the dynamic range and complicates absolute quantification. Count statistics, temporal sampling, attenuation and scatter correction, patient motion, and left ventricular input-function definition affect accuracy. The evidence base for isolated CMD is smaller and less standardized than for PET, and many published studies are single-center. Radiation exposure is generally higher than with contemporary PET protocols, although dose-reduction strategies are evolving. Dynamic CZT-SPECT is most reasonable when PET and quantitative CMR are unavailable, when a CZT platform with validated software and expertise is already established, or when flow quantification is being added to clinically indicated SPECT rather than ordered as a stand-alone experimental test.

## 7. Computed Tomography-Based Assessment

CCTA has a central role in CMD pathways because it can exclude obstructive epicardial disease and characterize plaque burden [39,40]. CCTA alone, however, does not measure microvascular function. A normal or non-obstructive CCTA can establish the anatomical prerequisite for considering isolated CMD, but symptoms and physiology remain unexplained until a functional test is performed [39,40]. CT-derived fractional flow reserve evaluates the physiological relevance of epicardial disease and should not be equated with microvascular assessment [39,40].

Stress CTP can be acquired as static or dynamic imaging. Static CTP captures myocardial iodine distribution at one time point, or a small number of them, and is interpreted as a relative perfusion map [39,41]. Dynamic CTP repeatedly samples contrast passage through the myocardium and can estimate MBF using time–attenuation curves [39,41]. Combining CCTA and CTP offers a one-session evaluation of coronary anatomy, plaque, and perfusion [39,41]. The CORE320 program established the incremental diagnostic value of combined CCTA and perfusion for obstructive CAD [39]. More recent dynamic CTP work has focused on quantitative flow, microvascular patterns, and physiologically informed modeling [39,42].

CT has high spatial resolution and can depict subendocardial perfusion, calcification, and coronary anatomy [39,41]. It may be particularly useful when CCTA is already indicated, and an integrated anatomic–functional examination can avoid sequential testing [39,41]. It is also an alternative when CMR is contraindicated. However, evidence specifically validating CT-derived MBF against invasive microvascular indices remains limited, and standardized CMD thresholds are not established [39,42]. Dynamic imaging entails radiation and iodinated contrast, and a rest–stress protocol may increase both [39,41,42]. Beam-hardening, cardiac motion, arrhythmia, contrast timing, and limited *z*-axis coverage can affect measurements [39,41]. Renal dysfunction, contrast allergy, and the need for repeated assessment are important limitations [39,41]. CTP should therefore be regarded as an emerging option for CMD rather than a first-line reference method.

New detector technology, whole-heart coverage, photon-counting CT, improved motion correction, and lower-dose dynamic protocols may make CT more competitive. The decisive question is not whether CT can generate an MBF map, but whether the values are reproducible, cross-vendor comparable, prognostically validated, and able to guide management beyond information already provided by CCTA and established perfusion modalities.

The principal characteristics, physiological signals, strengths, limitations, and potential clinical roles of the available non-invasive modalities are summarized in Table 2.

## 8. From Non-Invasive Physiology to Clinical Management

**Figure 1:**
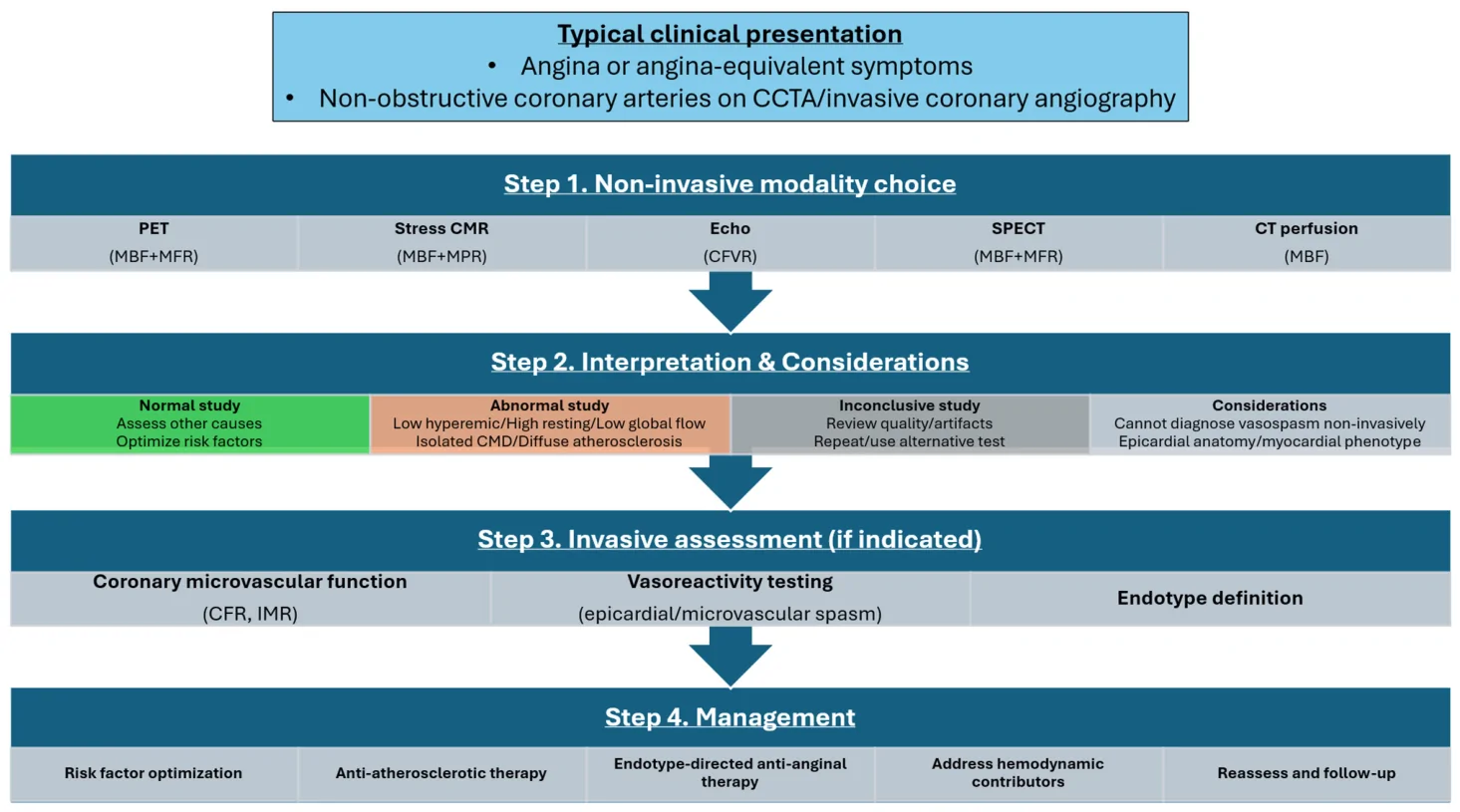
Approach to the non-invasive assessment of coronary microvascular dysfunction (CMD). CCTA, coronary computed tomography angiography; CFVR, coronary flow velocity reserve; CFR, coronary flow reserve; CMD, coronary microvascular dysfunction; CMR, cardiovascular magnetic resonance; CT, computed tomography; CZT, cadmium-zinc-telluride; IMR, index of microcirculatory resistance; INOCA, ischemia with non-obstructive coronary arteries; MBF, myocardial blood flow; MFR, myocardial flow reserve; MPR, myocardial perfusion reserve; PET, positron emission tomography; SPECT, single-photon emission computed tomography.

The clinical value of non-invasive coronary microvascular assessment lies not simply in detecting an abnormal flow index, but in clarifying the mechanism of symptoms, refining cardiovascular risk, and identifying patients who may benefit from further physiological investigation (Figure 1). Test selection should therefore begin with the clinical question and the available information on epicardial coronary anatomy. In patients with stable symptoms and an uncertain likelihood of obstructive CAD, CCTA is often an efficient first-line strategy because it can exclude flow-limiting stenosis and characterize atherosclerotic burden. Patients most likely to benefit from non-invasive coronary flow assessment include those with persistent angina or exertional dyspnea after obstructive epicardial CAD has been excluded or appears insufficient to explain symptoms, patients with discordance between symptoms and conventional relative perfusion imaging, individuals in whom diffuse or balanced coronary physiological impairment is suspected, and selected patients with myocardial disorders such as HFpEF or hypertrophic remodeling in whom quantitative perfusion may provide additional pathophysiological and prognostic information. The complementary physiological information provided by non-invasive and invasive coronary function testing is summarized in Table 3. Current European guidance supports the use of PET or stress CMR in relevant non-obstructive coronary syndromes and recommends invasive coronary function testing when symptoms persist despite non-obstructive anatomy and a definitive physiological diagnosis is required [40].

The therapeutic consequence of an abnormal flow study depends on the physiological pattern rather than on MFR alone. When reduced flow reserve is driven by reduced hyperemic MBF after adequate stress, and flow-limiting epicardial disease has been excluded, impaired vasodilator capacity is likely, and the optimization of cardiovascular risk factors and symptom-directed antianginal therapy are appropriate. Persistent symptoms may justify invasive assessment of CFR, microvascular resistance, and acetylcholine-mediated vasomotion to establish a more specific endotype. In contrast, a low MFR caused predominantly by elevated resting MBF should prompt assessment and correction of potential hemodynamic drivers such as tachycardia, hypertension, anemia, obesity, pain, or increased myocardial workload rather than automatic attribution to severe structural CMD.

Diffuse non-obstructive atherosclerosis represents a further important phenotype because reduced MFR in this setting reflects integrated epicardial and microvascular disease rather than isolated CMD. These patients should undergo intensive management of modifiable cardiovascular risk according to their overall atherosclerotic risk and plaque burden. Conversely, recurrent rest angina, circadian symptoms, or other clinical features suggesting vasospasm may warrant invasive acetylcholine testing even when vasodilator MBF and MFR are preserved, because conventional non-invasive vasodilator imaging cannot exclude epicardial or microvascular spasm.

Pharmacological therapy should therefore be phenotype-oriented whenever possible. In patients in whom invasive testing confirms microvascular angina associated with impaired CFR and/or increased microvascular resistance, beta blockers, calcium channel blockers, and renin–angiotensin system modulation may be considered according to the clinical setting, whereas calcium channel blockers form the cornerstone of therapy when a vasospastic endotype is demonstrated. Importantly, evidence that a treatment selected solely on the basis of a non-invasive MFR threshold improves cardiovascular outcomes remains insufficient. Thus, quantitative imaging should inform diagnosis, risk assessment, preventive intensity, and the need for further endotyping rather than act as an isolated prescription trigger.

Clinical vignette:

A 58-year-old woman with persistent exertional angina undergoes CCTA, demonstrating mild non-obstructive coronary atherosclerosis. PET shows normal relative perfusion but globally reduced hyperemic MBF (1.4 mL/min/g) with resting MBF of 0.9 mL/min/g and an MFR of 1.56. After confirmation of adequate vasodilator stress, the findings indicate impaired global vasodilator capacity but do not establish isolated CMD, because diffuse epicardial atherosclerosis may also contribute. Cardiovascular risk factors and antianginal therapy are optimized. Because symptoms remain limiting, invasive coronary function testing is performed and demonstrates preserved epicardial physiology, reduced CFR, increased microvascular resistance, and no acetylcholine-provoked spasm, supporting a structural microvascular endotype. Therapy can consequently be directed toward microvascular angina. Had acetylcholine instead demonstrated epicardial or microvascular spasm, a vasospastic treatment strategy would have been favored.

## 9. Future Directions and Unmet Needs

Despite substantial progress in non-invasive assessment of CMD, several barriers limit broader clinical implementation. Foremost is the need for greater standardization of acquisition, analysis, and reporting across platforms. Absolute MBF and MFR remain dependent on tracer, scanner, sequence, reconstruction method, software, and patient characteristics, limiting the applicability of universal diagnostic thresholds. Platform-specific reference ranges, ideally incorporating age, sex, and relevant comorbidities, together with automated quality-control procedures, will be important for improving reproducibility and cross-center comparability.

Technological advances may further expand access to quantitative perfusion imaging. Wider availability of longer-lived PET tracers, automated quantitative CMR perfusion mapping, dynamic CZT-SPECT, improved motion correction, whole-heart CT coverage, and photon-counting CT may increase the feasibility and precision of non-invasive flow assessment. However, technical capability alone is insufficient, and emerging approaches require prospective validation against established physiological standards and demonstration of incremental diagnostic and prognostic value beyond conventional anatomical and perfusion imaging.

The principal remaining clinical gap is whether imaging-defined physiological phenotypes can guide treatment and improve outcomes. Reduced flow reserve is prognostically informative, but it does not identify a unique mechanism and should not dictate a specific therapy itself. Future studies should therefore move beyond diagnostic accuracy toward prospective, phenotype-guided strategies that integrate absolute flow, coronary anatomy, myocardial phenotype, symptoms, and, when necessary, invasive coronary function testing. Demonstrating that such an approach improves symptoms, quality of life, healthcare utilization, or cardiovascular outcomes will be essential for establishing non-invasive CMD assessment as a treatment-guiding, rather than predominantly diagnostic and risk stratification, tool.

## 10. Conclusions

Non-invasive assessment of CMD has evolved from indirect detection of ischemia to quantitative characterization of coronary vasodilator function. PET provides the strongest validation and prognostic evidence, while quantitative perfusion CMR offers high-resolution flow mapping with comprehensive tissue characterization. Doppler echocardiography provides an accessible CFVR estimate, and MCE, dynamic CZT-SPECT, and stress CTP offer complementary or emerging approaches.

Clinically sound interpretation requires adequate stress, review of absolute stress and resting flow, exclusion or characterization of epicardial atherosclerosis, and consideration of scar, hypertrophy, hemodynamics, and technical quality. Non-invasive testing is most valuable when it confirms impaired vasodilator capacity, refines prognosis, and guides referral for invasive endotyping in patients with persistent symptoms. It cannot reliably diagnose vasospasm and should not be used as a stand-alone therapeutic target. Future progress depends on standardized acquisition and reporting, automated quality assurance, diverse reference populations, and prospective trials in physiology-defined endotypes.

## Figures and Tables

**Table 1:** Interpretation of non-invasive coronary flow measurements.

Flow Pattern	Principal Interpretation	Practical Implication
Normal rest flow + normal stress flow + preserved reserve	Preserved global vasodilator capacity	If typical symptoms persist, consider alternative mechanisms including vasospasm
Normal rest flow + reduced stress flow + reduced reserve	Impaired vasodilator capacity	Exclude inadequate stress, obstructive CAD and artifact Consider CMD and/or diffuse atherosclerosis
Elevated rest flow + preserved stress flow + reduced reserve	High-rest-flow phenotype	Assess heart rate, blood pressure, anemia, obesity, pain/anxiety and myocardial workload
Reduced rest and stress flow + apparently preserved reserve	Global low-flow phenotype; ratio may be misleading	Do not consider physiology normal on the basis of MFR alone; review absolute MBF, technical quality, hemodynamics and myocardial phenotype
Regional reduction in stress flow/reserve with concordant defect	Focal epicardial disease more likely	Reassess epicardial anatomy and lesion significance
Global reduction in stress flow/reserve without focal defect	Diffuse coronary circulatory dysfunction	Consider CMD, diffuse plaque and multivessel disease Integrate with CCTA/angiography
Reduced flow associated with scar, hypertrophy or abnormal myocardial phenotype	Reduced myocardial perfusion not necessarily due to primary CMD	Integrate tissue phenotype and underlying myocardial disease
Inadequate stress or technical artifact	Non-diagnostic/potentially false result	Correct technical or pharmacological issue before diagnosing CMD

CAD, coronary artery disease; CCTA, coronary computed tomography angiography; CMD, coronary microvascular dysfunction.

**Table 2:** Comparative characteristics of non-invasive modalities for assessment of coronary microvascular dysfunction.

Modality	Measurements	Typical Interpretive Range/Threshold Reliability	Major Strengths	Major Limitations	Most-Appropriate Clinical Role	Supporting Evidence
PET MPI	Absolute rest and stress MBF Global and regional MFR	MFR >2.0 commonly considered preserved; 1.7–2.0 mildly reduced; 1.2–<1.7 reduced; <1.2 severely reduced. These are approximate interpretive categories rather than universal biological thresholds and remain tracer-, model-, software-, and laboratory-dependent. Hyperemic MBF Commonly <1.7–2.3 mL/min/g depending on tracer/model.	Most extensively validated technique Reproducible absolute flow quantification Strong prognostic evidence Sensitive to diffuse and balanced flow reduction Rapid acquisition Robust image quality No iodinated or gadolinium-based contrast	Limited availability Tracer/generator cost Radiation Dependence on kinetic model/software and rigorous quality control Motion and attenuation–emission misregistration Cannot diagnose vasospasm	Preferred when accurate absolute flow quantification and risk stratification are priorities, particularly in suspected diffuse or multivessel coronary dysfunction, poor echocardiographic windows, renal dysfunction, or when CMR is unsuitable	Diagnostic: Adequate Prognostic: Adequate Treatment-guiding: Limited
Stress CMR	Absolute rest and stress MBF Myocardial perfusion reserve Semiquantitative MPRI Pixel-wise perfusion mapping	MPR < ~1.5–2.0 has commonly been considered abnormal; no universal cross-platform threshold.	High spatial resolution Sensitive to subendocardial abnormalities No ionizing radiation Simultaneous assessment of ventricular function, scar, fibrosis, edema, inflammation, and cardiomyopathic phenotypes	Incomplete cross-platform standardization Gadolinium required for conventional perfusion Dark-rim and motion artifacts Arrhythmia/breath-holding limitations Contraindicated or difficult with some devices/claustrophobia Scanner/expertise availability	Particularly useful when CMD assessment must be integrated with myocardial tissue characterization, including suspected cardiomyopathy, myocarditis, scar, HFpEF, or hypertrophic remodeling	Diagnostic: Adequate Prognostic: Adequate Treatment-guiding: Limited
Transthoracic Doppler echocardi-ography	Rest and hyperemic CFVR (usually in distal LAD)	CFVR ≤ 2.0 commonly abnormal; thresholds up to 2.5 used depending on protocol/population.	Widely accessible Inexpensive No radiation No nephrotoxic contrast Repeatable May be incorporated into vasodilator stress echocardiography	Operator dependent Technically demanding Usually limited to LAD Velocity rather than absolute tissue-flow measurement Affected by acoustic window, vessel stenosis, heart rate, blood pressure, collaterals, and stress adequacy	Practical first-line or adjunctive physiological assessment in experienced echocardiography laboratories when LAD physiology is clinically informative	Diagnostic: Moderate Prognostic: Moderate Treatment-guiding: Limited
Myocardial contrast echocardi-ography	Microvascular blood volume and replenishment velocity (their product approximates myocardial blood flow)	No sufficiently validated universal CMD-specific threshold.	Tissue-level assessment of the entire myocardium No ionizing radiation Can demonstrate diffuse/subendocardial perfusion abnormalities	Acoustic attenuation/artifacts Dependence on microbubble destruction–replenishment methodology and expertise Limited standardization and outcome validation Contrast availability/reimbursement issues	Specialized alternative in experienced centers, particularly when tissue-level echocardiographic perfusion assessment is desired	Diagnostic: Limited Prognostic: Limited Treatment-guiding: Very limited
Dynamic CZT-SPECT	Global and regional absolute MBF and MFR derived from dynamic first-pass tracer imaging	MFR ≤ 2.0 frequently used; highly platform dependent.	Extends quantitative flow assessment to the more widely available SPECT infrastructure Simultaneous conventional perfusion assessment	Non-linear technetium tracer extraction at high flow Smaller evidence base than PET Limited standardization Count/statistical and input-function limitations Radiation exposure	Reasonable where PET/CMR are unavailable and a validated CZT platform with appropriate expertise is established	Diagnostic: Moderate Prognostic: Limited Treatment-guiding: Very limited
Stress CT perfusion	Static relative myocardial perfusion or dynamic quantitative MBF derived from time–attenuation curves	No sufficiently validated universal CMD-specific threshold.	High spatial resolution Simultaneous assessment of coronary anatomy, plaque burden, calcification, and myocardial perfusion Potential one-session anatomical–functional evaluation	Ionizing radiation Iodinated contrast Limited validation against invasive microvascular indices Beam-hardening/motion artifacts Arrhythmia and coverage limitations Problematic in renal dysfunction or contrast allergy	Emerging modality, most useful when integrated with clinically indicated CCTA rather than as a routine stand-alone CMD test	Diagnostic: Moderate Prognostic: Moderate Treatment-guiding: Limited

CFVR, coronary flow velocity reserve; CMR, cardiovascular magnetic resonance; CT, computed tomography; CZT, cadmium-zinc-telluride; LAD, left anterior descending coronary artery; MBF, myocardial blood flow; MCE, myocardial contrast echocardiography; MFR, myocardial flow reserve; MPR, myocardial perfusion reserve; MPRI, myocardial perfusion reserve index; PET, positron emission tomography; SPECT, single-photon emission computed tomography.

**Table 3:** Complementary roles of non-invasive and invasive coronary function testing.

Approach	Physiological Measurement	Main Information Provided	Limitations
PET/CMR MFR or MPR	Rest and hyperemic myocardial blood flow and their ratio	Global/regional coronary vasodilator capacity; identifies reduced hyperemic flow, high-rest-flow and global low-flow patterns	Cannot distinguish isolated CMD from diffuse epicardial disease; cannot diagnose epicardial or microvascular spasm
Doppler CFVR	Hyperemic-to-rest coronary flow velocity ratio, usually in the LAD	Vasodilator capacity in the interrogated coronary territory	Does not measure absolute tissue MBF or microvascular resistance; cannot diagnose vasospasm
Invasive CFR	Hyperemic-to-rest coronary blood flow ratio	Invasive assessment of overall coronary vasodilator reserve	Reduced CFR is not specific for increased microvascular resistance and does not identify vasospasm
Invasive IMR/HMR	Hyperemic microvascular resistance	Identifies increased microvascular resistance, supporting a structural/resistance-related CMD endotype	Does not assess endothelium-dependent vasomotion or diagnose vasospasm
Acetylcholine testing	Endothelium-dependent coronary vasomotor response	Identifies epicardial spasm, microvascular spasm and abnormal endothelial vasomotion	Does not quantify adenosine-mediated vasodilator reserve in the same manner as CFR/MFR

CFR, coronary flow reserve; CFVR, coronary flow velocity reserve; CMR, cardiovascular magnetic resonance; HMR, hyperemic microvascular resistance; IMR, index of microcirculatory resistance; LAD, left anterior descending coronary artery; MBF, myocardial blood flow; MFR, myocardial flow reserve; MPR, myocardial perfusion reserve; PET, positron emission tomography.

## Data Availability

No new data were created or analyzed in this study. Data sharing is not applicable to this article.
